# Supplementary figures and images for: A task-general connectivity model reveals variation in convergence of cortical inputs to functional regions of the cerebellum
Source: eLife. 2023 Apr 21;12:e81511. doi: 10.7554/eLife.81511 (PMC10129326; doi:10.7554/eLife.81511)

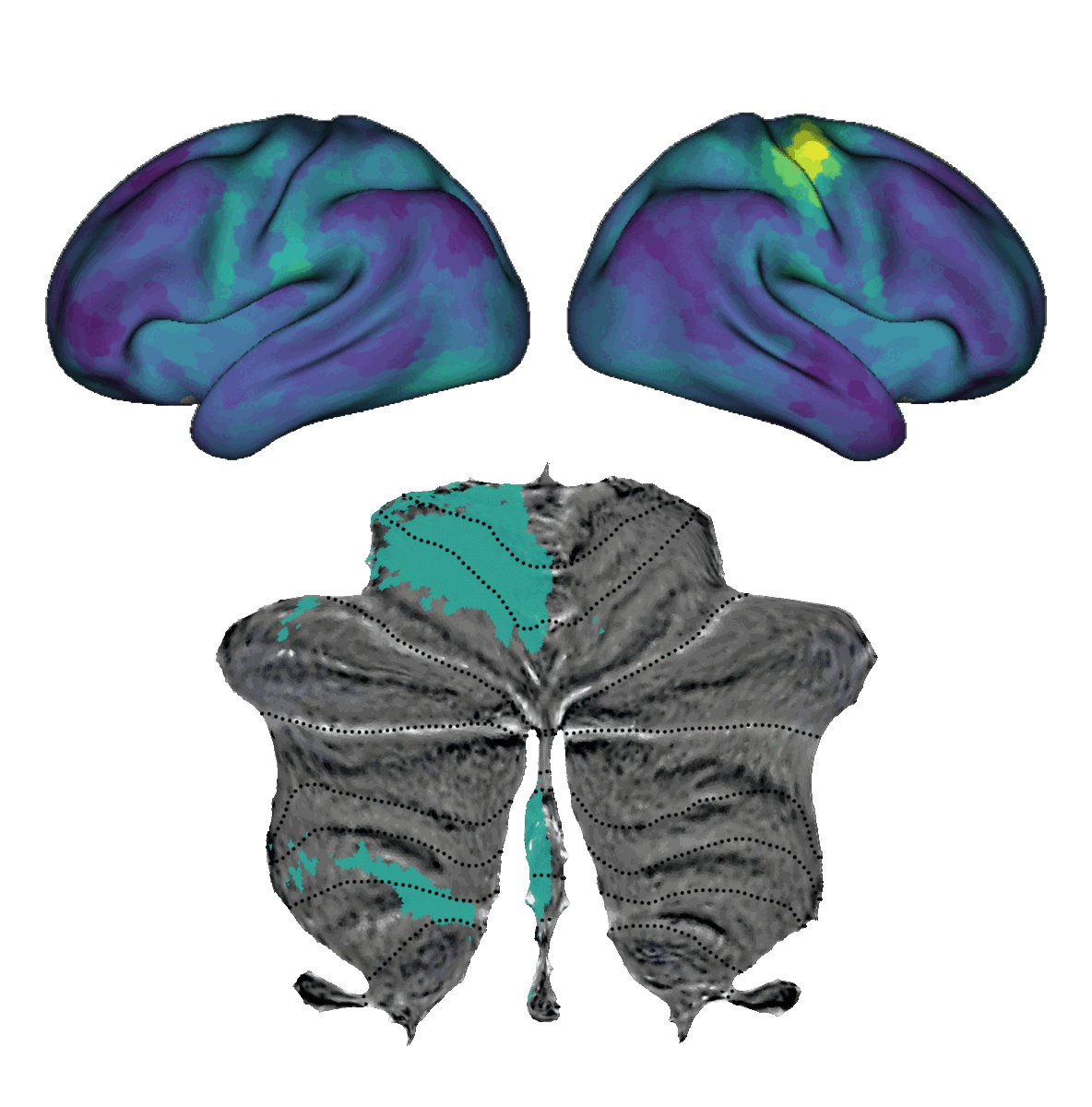

Supplement: Supplementary file 4 [file elife-81511-fig3-video1.gif]

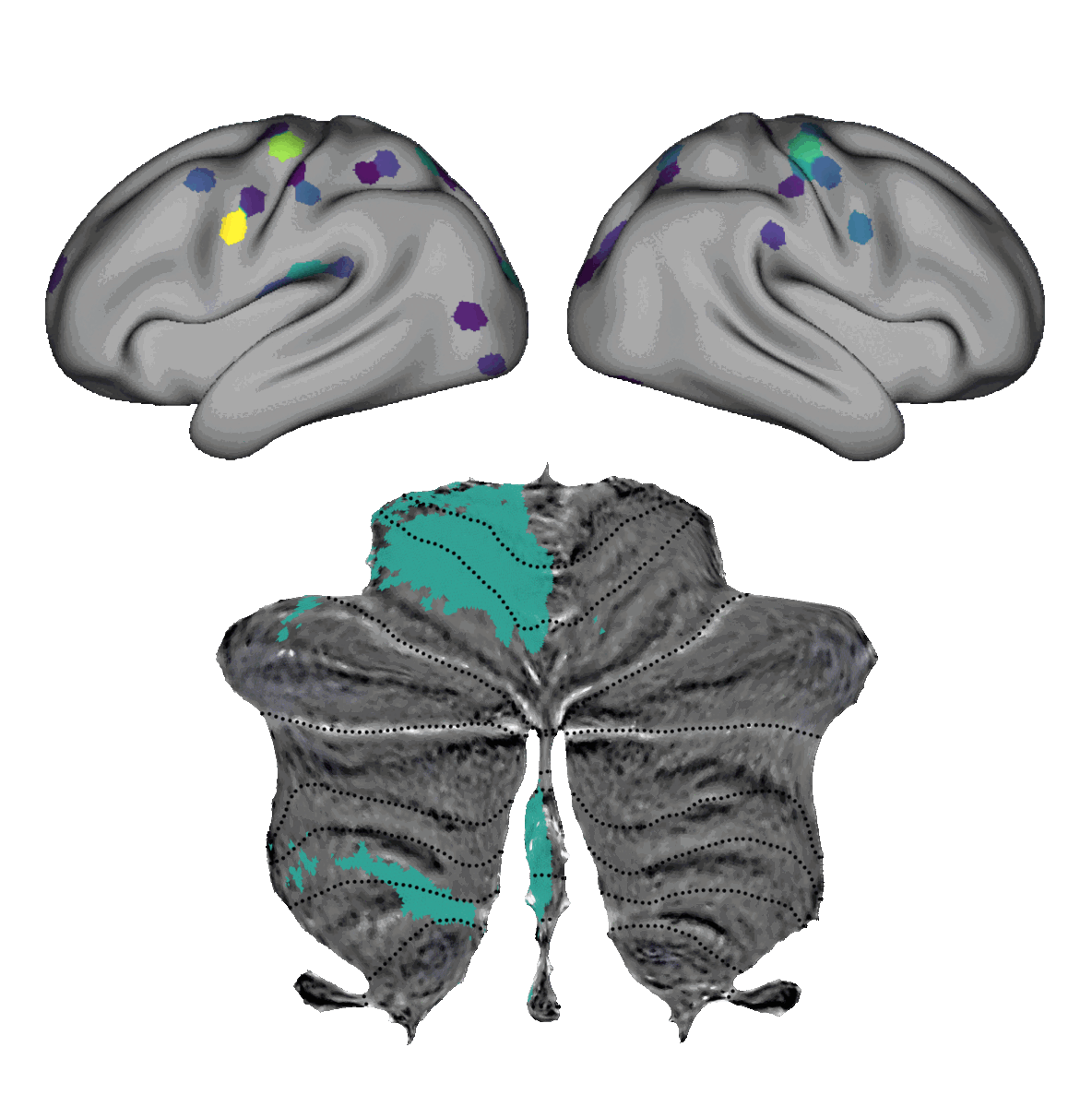

Supplement: Supplementary file 5 [file elife-81511-fig3-video2.gif]
